# Analysis of Spounaviruses as a Case Study for the Overdue Reclassification of Tailed Phages

**DOI:** 10.1093/sysbio/syz036

**Published:** 2019-05-25

**Authors:** Jakub Barylski, François Enault, Bas E Dutilh, Margo BP Schuller, Robert A Edwards, Annika Gillis, Jochen Klumpp, Petar Knezevic, Mart Krupovic, Jens H Kuhn, Rob Lavigne, Hanna M Oksanen, Matthew B Sullivan, Ho Bin Jang, Peter Simmonds, Pakorn Aiewsakun, Johannes Wittmann, Igor Tolstoy, J Rodney Brister, Andrew M Kropinski, Evelien M Adriaenssens

**Affiliations:** 1 Department of Molecular Virology, Institute of Experimental Biology, Faculty of Biology, Adam Mickiewicz University in Poznań, Collegium Biologicum - Umultowska 89, 61-614 Poznań, Poland; 2 Université Clermont Auvergne, CNRS, LMGE, F-63000 Clermont-Ferrand, France; 3 Theoretical Biology and Bioinformatics, Department of Biology, Science for Life, Utrecht University, Padualaan 8, 3584 CH, Utrecht, The Netherlands; 4 Centre for Molecular and Biomolecular Informatics, Radboud Institute for Molecular Life Sciences, Radboud University Medical Centre, Geert Grooteplein 28, 6525 GA, Nijmegen, The Netherlands; 5 Department of Biology, San Diego State University, 5500 Campanile Drive, San Diego, CA 92182, USA; 6 Department of Computer Science, San Diego State University, 5500 Campanile Drive, San Diego, CA 92182, USA; 7 Laboratory of Food and Environmental Microbiology, Université Catholique de Louvain, Croix du Sud 2-L7.05.12, 1348 Louvain-la-Neuve, Belgium; 8 Institute of Food, Nutrition and Health, ETH Zurich, Schmelzbergstrasse 7, 8092 Zurich, Switzerland; 9 Department of Biology and Ecology, Faculty of Sciences, University of Novi Sad, Novi Sad, Serbia; 10 Unité Biologie Moléculaire du Gène chez les Extrêmophiles, Institut Pasteur, 25 rue du Dr. Roux, 75015 Paris, France; 11 Integrated Research Facility at Fort Detrick, Division of Clinical Research, National Institute of Allergy and Infectious Diseases, National Institutes of Health, B-8200 Research Plaza, Fort Detrick, Frederick, MD 21702, USA; 12 Laboratory of Gene Technology, Department of Biosystems, KU Leuven, Kasteelpark Arenberg 21 - box 2462, 3001 Leuven, Belgium; 13 Molecular and Integrative Biosciences Research Programme, Faculty of Biological and Environmental Sciences, University of Helsinki, P.O. Box 56 (Viikinkaari 9B), 00014 Helsinki, Finland; 14 Department of Microbiology, The Ohio State University, 496 W 12thAvenue, Columbus, OH 43210, USA; 15 Department of Civil, Environmental, and Geodetic Engineering, The Ohio State University, 496 W 12thAvenue, Columbus, OH 43210, USA; 16 Nuffield Department of Medicine, University of Oxford, Peter Medawar Building, South Parks Road, Oxford OX1 3SY, UK; 17 Department of Microbiology, Faculty of Science, Mahidol University, Bangkok 10400, Thailand; 18 Leibniz-Institut DSMZ—German Collection of Microorganisms and Cell Cultures, Inhoffenstr. 7B, 38124 Braunschweig, Germany; 19 National Center for Biotechnology Information, National Library of Medicine, National Institutes of Health, 8600 Rockville Pike, Bethesda MD 20894, USA; 20 Department of Food Science, University of Guelph, Guelph, Ontario, Canada; 21 Department of Pathobiology, University of Guelph, 50 Stone Road E, Guelph, Ontario N1G 2W1, Canada; 22 Department of Functional & Comparative Genomics, Institute of Integrative Biology, University of Liverpool, Biosciences Building, Crown Street, Liverpool L69 7ZB, UK; 23 Gut Microbes & Health Institute Strategic Programme, Quadram Institute Bioscience, Norwich Research Park, James Watson Road, Norwich NR4 7UQ Norwich, UK

**Keywords:** Caudovirales, Herelleviridae, phylogenetics, phylogenomics, spounavirus, virus classification, virus taxonomy

## Abstract

Tailed bacteriophages are the most abundant and diverse viruses in the world, with genome sizes ranging from 10 kbp to over 500 kbp. Yet, due to historical reasons, all this diversity is confined to a single virus order—*Caudovirales*, composed of just four families: *Myoviridae*, *Siphoviridae, Podoviridae*, and the newly created *Ackermannviridae* family. In recent years, this morphology-based classification scheme has started to crumble under the constant flood of phage sequences, revealing that tailed phages are even more genetically diverse than once thought. This prompted us, the Bacterial and Archaeal Viruses Subcommittee of the International Committee on Taxonomy of Viruses (ICTV), to consider overall reorganization of phage taxonomy. In this study, we used a wide range of complementary methods—including comparative genomics, core genome analysis, and marker gene phylogenetics—to show that the group of Bacillus phage SPO1-related viruses previously classified into the *Spounavirinae* subfamily, is clearly distinct from other members of the family *Myoviridae* and its diversity deserves the rank of an autonomous family. Thus, we removed this group from the *Myoviridae* family and created the family *Herelleviridae*—a new taxon of the same rank. In the process of the taxon evaluation, we explored the feasibility of different demarcation criteria and critically evaluated the usefulness of our methods for phage classification. The convergence of results, drawing a consistent and comprehensive picture of a new family with associated subfamilies, regardless of method, demonstrates that the tools applied here are particularly useful in phage taxonomy. We are convinced that creation of this novel family is a crucial milestone toward much-needed reclassification in the *Caudovirales* order.

By the end of 2018, nearly 8000 complete tailed phage genomes were published online and a further 22,000 partial genomes were stored in databases gathered under the umbrella of the International Nucleotide Sequence Database Collaboration (Karsch-Mizrachi et al. 2012; O’Leary et al. 2016). The classification of this massive group is the formal responsibility of the Bacterial and Archaeal Viruses Subcommittee of the International Committee on the Taxonomy of Viruses (ICTV). In recent years, we (the Subcommittee) have focused on classifying newly described phages into species and genera (Lavigne et al. 2008, 2009; Adriaenssens et al. 2015; Krupovic et al. 2016; Adriaenssens et al. 2017). However, once our attention shifted toward higher order relationships, we found that the ranks currently used in phage taxonomy (species, genus, subfamily, family, and order) are no longer sufficient for the description of phage diversity. The limitation is particularly acute in the case of the order *Caudovirales*—arguably the most abundant and heterogeneous group of viruses (Paez-Espino et al. 2016; Roux et al. 2016; Nishimura et al. 2017a). Indeed, the diversity of caudoviruses surpasses that of any other virus taxon. A recent analysis of the gene content of the dsDNA virosphere demonstrated that the global network of dsDNA viruses consists of at least 19 modules, 11 of which correspond to caudoviruses (Iranzo et al. 2016). Each of the eight remaining modules encompasses one or more families of eukaryotic or archaeal viruses. Consequently, each of the 11 caudovirus modules could be considered a separate family. Despite this remarkable diversity, the vast majority of caudoviruses is classified into three families *Myoviridae*, *Podoviridae*, and *Siphoviridae*, which were historically established on morphological features, forming an artificial classification ceiling. These observations prompted us to work on the update of current taxonomic order within the *Caudovirales* order.

As an initial step of this major reclassification of the tailed phages, we, the members of the Subcommittee proposed creation of two novel families corresponding to distinct modules revealed in the abovementioned gene-sharing network analyses (Iranzo et al. 2016; Bolduc et al. 2017a). The first of these, named *Ackermannviridae*, encompasses phages related to *Salmonella virus ViI* that were formerly assigned to the genus *Viunalikevirus* (Adriaenssens et al. 2012, 2018). In the present work, we focus on the second new family, named *Herelleviridae*. The phages belonging to this new family are large myoviruses related to the Bacillus phage SPO1, Staphylococcus phage Twort, Staphylococcus phage K, Listeria phage P100, and Enterococcus phage $\varphi$EF24C. Most of these viruses were previously grouped in the *Spounavirinae* subfamily or recognized as related to it. When this subfamily was first devised (Lavigne et al. 2009), the unifying characteristics of its members included: the hosts belong to the bacterial phylum *Firmicutes*; strictly virulent lifestyle; myovirion morphology (i.e., icosahedral capsid and long contractile tail); terminally redundant, nonpermuted dsDNA genome of 127–157 kbp in length; and “considerable amino acid similarity” (Klumpp et al. 2010). The strictly virulent lifestyle of these viruses has been somewhat disputed (Schuch and Fischetti 2009; Yuan et al. 2015) but still remains a rule of thumb for inclusion into the taxon. Since the initial description of the subfamily, the number of its members has grown significantly, and its taxonomic structure has been contested several times (Klumpp et al. 2010; Barylski et al. 2014; Iranzo et al. 2016; Krupovic et al. 2016; Bolduc et al. 2017a; Adriaenssens et al. 2017). Thus, we wanted not only to delineate a new family but also resolve its internal structure.

Unfortunately, there is no one-size-fits-all method for the classification of viruses at all taxonomic ranks. Virus taxonomy has always suffered from the lack of universal marker genes that could be used for phylogenetic reconstruction of the evolutionary relationships. Additionally, differing mutation rates between viral lineages, horizontal gene transfer, and genomic mosaicism limit usefulness of many of the available phylogenetic and phylogenomic methods that have become the gold standard in evolutionary biology (Davidson et al. 2015; Meier-Kolthoff and Göker 2017). Thus, our strategy for reclassification included a plethora of classification tools that employ very different approaches. Our analyses ranged from coarse-grained, high-throughput, holistic clustering methods where similarity is computed from comparison of all viral genes [vContact, GRAViTy (Bolduc et al. 2017a; Aiewsakun et al. 2018; Aiewsakun and Simmonds 2018)] to detailed genome and proteome comparisons [Victor, Dice, GOAT and Phage Proteomic Tree (Rohwer and Edwards 2002; Mizuno et al. 2013; Meier-Kolthoff and Göker 2017)] and individual gene phylogenies [IQtree (Nguyen et al. 2015)]. This multifaceted approach allowed us to gradually descend from the definition of the new family to the study of its internal structure. Interestingly, despite the diversity of the applied methods their results turned out to be complementary and predominantly concordant. All methods painted a robust picture of the new family as a distinct and diverse taxon and supported the same general scheme for its structure (Table 1).

**Table 1.** New classification of the 93 spounaviruses and spouna-like viruses in the new family *Herelleviridae*$^{a}$

Family	Subfamily	Genus$^{a}$	Species$^{b}$
*Herelleviridae*	*Bastillevirinae*	*Agatevirus*	*Bacillus virus Agate*, *Bacillus virus Bobb*, *Bacillus virus Bp8pC* (Bp8p-T)
		*Bequatrovirus* (formerly *B4virus*)	*Bacillus virus AvesoBmore*, *Bacillus virus B4* (B5S), *Bacillus virus Bigbertha, Bacillus virus Riley, Bacillus virus Spock, Bacillus virus Troll*
		*Bastillevirus*	*Bacillus virus Bastille, Bacillus virus CAM003, Bacillus virus Evoli, Bacillus virus HoodyT*
		*Caeruleovirus* (formerly *Bc431virus*)	*Bacillus virus Bc431*, *Bacillus virus Bcp1*, *Bacillus virus BCP82*, *Bacillus virus JBP901*
		*Nitunavirus* (formerly *Nit1virus*)	*Bacillus virus Grass*, *Bacillus virus NIT1*, *Bacillus virus SPG24*
		*Tsarbombavirus*	*Bacillus virus BCP78* (BCU4), *Bacillus virus TsarBomba*
		*Wphvirus*	*Bacillus virus BPS13*, *Bacillus virus Hakuna*, *Bacillus virus Megatron* (Eyuki), *Bacillus virus WPh*, *Bacillus virus BPS10C*
		Unassigned	*Bacillus virus Mater*, *Bacillus virus Moonbeam*, *Bacillus virus SIOphi*
	*Brockvirinae*	*Kochikohdavirus*	*Enterococcus virus ECP3*, *Enterococcus virus EF24C* (phiEFC24C-P2), *Enterococcus virus EFLK1*
		Unassigned	*Enterococccus virus EFDG1*
	*Jasinskavirinae*	*Pecentumvirus* (formerly *P100virus*)	*Listeria virus A511*, *Listeria virus P100*, *Listeria virus List36*, *Listeria virus LMSP25* (LMTA-57, LMTA-94), *Listeria virus LMTA148*, *Listeria virus LMTA34*, *Listeria virus LP048*, *Listeria virus LP064* (LP-125), *Listeria virus LP083-*2 (LP-124), *Listeria virus AG20*, *Listeria virus WIL1*
	*Spounavirinae*	*Siminovitchvirus* (formerly *Cp51virus*)	*Bacillus virus CP51*, *Bacillus virus JL*, *Bacillus virus Shanette*
		*Okubovirus* (formerly *Spo1virus*)	*Bacillus virus Camphawk*, *Bacillus virus SPO1*
	*Twortvirinae*	*Kayvirus*	*Staphylococcus virus G1*, *Staphylococcus virus G15*, *Staphylococcus virus JD7*, *Staphylococcus virus K*, *Staphylococcus virus MCE2014*, *Staphylococcus virus P108*, *Staphylococcus virus Rodi*, *Staphylococcus virus S253*, *Staphylococcus virus S25-4*, *Staphylococcus virus SA12*, *Staphylococcus virus Sb1* (676Z, A3R, A5W, Fi200W, IME-SA1, IME-SA118, IME-SA119, IME-SA2, ISP, MSA6, P4W, SA5, Staph1N, Team1)
		*Silviavirus*	*Staphylococcus virus Remus* (Romulus), *Staphylococcus virus SA11*
		*Sepunavirus* (formerly *Sep1virus*)	*Staphylococcus virus IPLAC1C*, *Staphylococcus virus SEP1*
		*Twortvirus*	*Staphylococcus virus Twort*
	Unassigned	Unassigned	*Lactobacillus virus Lb338*
	Unassigned	Unassigned	*Lactobacillus virus LP65*
	Unassigned	Unassigned	*Brochothrix virus A9*

$^{a}$Genera were renamed in 2018, taxonomy proposal 2018.007B.

$^{b}$The species listed here represent the 93 genome data set on which all analyses have been performed. Phage isolates at the subspecies or strain level are indicated between brackets.

We emphasize that this reclassification is an essential step in the larger revision of the taxonomy of the order *Caudovirales*. The final goal of our group is a novel system that appropriately accommodates the genomic diversity of prokaryotic viruses and is consistent with taxonomy of eukaryotic viruses (Aiewsakun et al. 2018; Simmonds and Aiewsakun 2018).

## Materials and Methods

For brevity and clarity’s sake, only the basic principles of previously published methods are summarized in the following section. A detailed description of each method used in this study can be found in Supplementary File 1 available on Dryad at http://dx.doi.org/10.5061/dryad.106q6g6.

### Creation of the “Herelleviridae” Data Set

Genome sequences of known spounaviruses were retrieved from the GenBank or (preferably) RefSeq databases based on literature data, and taxonomic classifications provided by the ICTV and the National Center for Biotechnology Information (NCBI). Records representing genomes of candidate spouna-related viruses were retrieved by searching the same databases with the tBLASTn algorithm (Altschul et al. 1990) using as queries terminase and major capsid proteins of type isolates of the original subfamily (Brister et al. 2015). After manual curation, the search yielded a set of 93 virus genomes (Supplementary Table S1.1 available on Dryad), which were reannotated using PROKKA (Seemann 2014) and used in the following analyses.

To conduct interfamilial comparisons, we compiled an additional genome set including well-described viruses from the ICTV 2016 Master Species List 31V1.1 and Virus Metadata Resource (Supplementary Table S1.2 available on Dryad).

All original genome sequences are available from NCBI (accession number information listed in Supplementary Table S1 available on Dryad) and the reannotated genomes are available from Github (github.com/evelienadri/herelleviridae).

### Definition of the New Herelleviridae Family Within the dsDNA Virosphere

We examined whether or not the family, *Herelleviridae*, is a clearly distinct group of viruses within the dsDNA phages, by using two cutting-edge virus clustering tools capable of discerning relations even between divergent taxa.

Using vConTACT v2.0, we constructed a monopartite network of viral genomes by clustering gene families based on BLAST hits between their protein products as previously described (Bolduc et al. 2017a; Jang et al. 2019). In this framework, similarities between pairs of genomes were calculated as a function of the shared protein families. The network was visualized with Cytoscape (version 3.5.1; http://cytoscape.org/) with genomes sharing more proteins clustered more closely together (detailed information in Supplementary File 1 available on Dryad).

The second method used is ‘Genome Relationships Applied to Virus Taxonomy’ or GRAViTy [GitHub: Paiewsakun/GRAViTy (Aiewsakun et al. 2018; Aiewsakun and Simmonds 2018)]. This framework created a dendrogram of viruses, based on protein profile hidden Markov models of the predicted gene products and genome organization models calculated into a composite generalized Jaccard (CGJ) score representing the difference between two viruses on a scale from 1 to 0 (detailed information in Supplementary File 1 available on Dryad).

We also investigated the clustering of the family within the *Caudovirales* order on the VIPtree server (Nishimura et al. 2017b), which uses the Phage Proteomic Tree approach described below and detailed in in Supplementary File 1 available on Dryad.

### Exploration of the Intrafamilial Relationship

After demarcation of the family, we proceeded with analysis of its internal structure, using the defined set of 93 genomes described above. In the process, we compared a collection of the classification tools, gathering the phylogenetic signal from the different types of data (whole genome sequences, complete proteomes, marker genes, and gene order).

### Genome-Based Analyses

Nucleotide sequence-based grouping of phages was conducted using VICTOR (Virus Classification and Tree Building Online Resource), a Genome-BLAST Distance Phylogeny (GBDP) method (Meier-Kolthoff et al. 2014; Meier-Kolthoff and Göker 2017). The program calculates intergenomic distances from BLAST+ hits using GBDP (including 100 pseudobootstrap replicates) and used them to infer a balanced minimum evolution tree with branch support via FASTME including subtree pruning and regrafting postprocessing (for details of the algorithm design, see Meier-Kolthoff et al. 2014; Meier-Kolthoff and Göker 2017). The analysis was conducted under settings recommended for prokaryotic viruses.

To reevaluate and interpret results of the VICTOR clustering, we compared the genome sequences using the Gegenees tool with default parameters (Camacho et al. 2009; Ågren et al. 2012). The program calculated symmetrical identity (SI) scores for each pairwise comparison based on BLASTn hits and a genome length.

To check if the translated local alignment of the whole genomes will be more sensitive to a phylogenetic signal at higher taxonomic ranks, we followed the Dice methodology proposed previously (Mizuno et al. 2013). The Dice score was calculated based on all reciprocal tBLASTx hits between pairs of genomes with $\ge$30% identity, alignment length $\ge$30 amino acids, and $E$-value $\le$0.01. Pairs of scores were used to construct a distance matrix, which in turn was converted to the final tree using the BioNJ algorithm (Gascuel 1997). Again, to evaluate and interpret this result, we calculated SI scores between all translated genome sequences using Gegenees. This time, we applied tBLASTx as the alignment algorithm with the other settings left on default values.

### Proteome-Based Analyses

The Phage Proteomic Tree was constructed as described previously (Rohwer and Edwards 2002). In brief, the protein sequences were extracted and clustered using BLASTp. These clusters were refined by Smith–Waterman alignment using CLUSTALW version 2 (Larkin et al. 2007). Alignments were scored using open-source PROTDIST from the phylogeny inference package (PHYLIP) (Felsenstein 1989). Alignment scores were converted to distances as described in Rohwer and Edwards (2002), and the distances thus obtained were used to generate the final tree using the neighbor joining algorithm.

### Identification of Protein Clusters

In order to comprehensively define the gene content in *herellevirus* genomes, we applied two independent, yet complementary methods of identifying orthologous clusters.

An initial set of orthologous protein clusters (OPCs) was constructed using the GET_HOMOLOGUES software suite, which utilizes several independent clustering methods (Contreras-Moreira and Vinuesa 2013). To capture as many evolutionary relationships as possible, a greedy COGtriangles algorithm (Kristensen et al. 2010) was applied with a 50% sequence identity threshold, 50% coverage threshold, and an $E$-value cutoff equal to 1e-10. The results were converted into an orthologue matrix with the “compare_clusters” script (part of the GET_HOMOLOGUES suite) (Felsenstein 1989).

A second method was based on assignment of the genes to a predefined pVOG (prokaryotic Virus Orthologous Group) set described previously (Grazziotin et al. 2017) and available at http://dmk-brain.ecn.uiowa.edu/pVOGs/. In brief, protein-coding genes in the 93 analyzed genomes were identified using Prodigal V2.6.3 in anonymous mode (Hyatt et al. 2010). Then, the gene products were assigned to the respective orthologue group by HMMsearch ($E$-value $<$10$^{-2}$) against the database of Hidden Markov Models (HMMs) created for every of 9518 pVOG alignments using HMMbuild of HMMer v3.1b2 (Finn et al. 2011).

### Analysis of Gene Synteny

To investigate a genomic synteny-based classification signal, we implemented a method developed at the University of Utrecht, a gene order-based metric built on dynamic programming, the Gene Order Alignment Tool (GOAT, Schuller et al.: Python scripts are available on request, manuscript in preparation). The tool used the pVOG assignments described above to generate a synteny profile of every genome (in fact, this pVOGs methodology is integral part of the GOAT pipeline).

The algorithm accounted for gene replacements and low similarity between genes by using an all-vs-all similarity matrix between pVOG pairs based on HMM–HMM similarity (HH-suite 2.0.16) (Söding et al. 2005). Distant HHsearch similarity scores between protein families were calculated as the average of reciprocal hits and used as substitution scores in the gene order alignment. The GOAT algorithm identified the optimal gene order alignment score between two virus genomes by implementing semiglobal dynamic programming alignment based only on the order of pVOGs identified on every virus genome. To account for virus genomes being cut at arbitrary positions during sequence assembly, the gene order was transmuted at all possible positions and in both sense and antisense directions in search of the optimal alignment score. The optimal GOAT alignment score GAB between every pair of virus genomes A and B was converted to a distance DAB as follows:

$$
\begin{align*}
{\rm DAB} = 1 - \frac{GAB + GBA}{GAA + GBB}
\end{align*}
$$

in which GAB and GBA represent the optimal GOAT score between A and B, and B and A, respectively, while GAA and GBB represent the GOAT scores of the self-alignments of A and B, respectively. This pairwise distance matrix was converted to a tree with BioNJ (Gascuel 1997).

### Marker Protein Phylogenies

Based on the OPC and pVOG clusters defined above, which respectively identified 14 and 38 core protein clusters (Supplementary Table S2 available on Dryad), we chose 10 consistently-predicted protein groups (encoded by genes with well-defined boundaries and without introns) for inclusion as phylogenetic marker. The selected clusters included: DNA helicase cluster, tail sheath protein, two different groups of virion proteins (including the major capsid protein cluster), and six clusters with no known function. The members of these clusters were aligned using Clustal Omega with default parameters (Sievers et al. 2011). The resulting alignments were analyzed with the IQ-TREE pipeline, which includes the ModelFinder tool that determines the most suitable model of sequence evolution for the alignment, the main algorithm that constructs a maximum-likelihood tree and ultrafast bootstrap (UFBOOT)—an UFBOOT subroutine that calculates the support of the branches (Nguyen et al. 2015; Chernomor et al. 2016; Kalyaanamoorthy et al. 2017; Hoang et al. 2018). The same program was used to generate the approximation of the “species tree” based on the concatenated alignments of all markers. In this case, the partitioned model of the alignment was also calculated using the ModelFinder module of IQ-TREE and the analysis was run in 100 replicates to select the final tree with best log-likelihood score.

### Visualization and Comparison of the Results

All trees were rooted at Brochothrix phage A9—a phage that consistently appeared as a distant outlier in all obtained topologies (to facilitate comparisons) and visualized using Geneious tree viewer. The taxon coloring and the legend was added using Inkscape 0.92.3 with no distortion of topology, branch lengths, or support.

Topological distances between different trees were calculated as Robinson–Foulds metrics (Robinson and Foulds 1981) with IQ-TREE and detected differences were visualized as tanglegrams generated using Neighbor Net-based heuristics in Dendroscope 3.5.9 (Huson and Scornavacca 2012).

## Results

### Definition of the Candidate “Herelleviridae” Family

**Figure 1.**
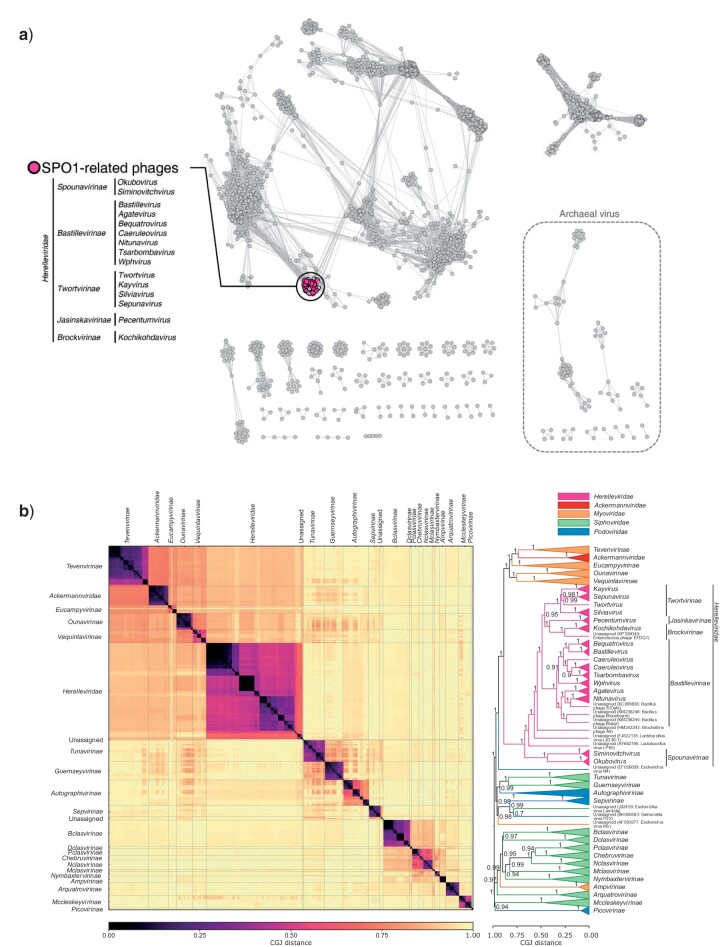
a) Network representation of predicted protein content similarity of dsDNA viruses generated with vConTACT v2.0. Viruses are represented as circles (nodes) connected with each other (edges) based on a significant number of shared protein clusters, with more similar genomes displayed closer together on the network. The genomes belonging to the new family *Herelleviridae* are indicated with a circle. Genomes previously assigned to the subfamily *Spounavirinae* are indicated in pink. b) Clustering of dsDNA bacteriophages that possess subfamily assignments in the order *Caudovirales* generated with GRAViTy, darker colors in the heatmap represent higher degrees of similarity between genomes. The phages are clustered using UPGMA into a dendrogram, showing bootstrap values (100 pseudoreplicates) on each branch.

Recently, several studies have shown the paraphyly of the families constituting the order *Caudovirales* (Iranzo et al. 2016; Bolduc et al. 2017a; Aiewsakun et al. 2018). We created a monopartite network of all dsDNA viruses in the NCBI RefSeq using vConTACT v2.0 (Bolduc et al. 2017a, Bolduc et al. under revision) showing the phages related to SPO1 as a clearly defined, interrelated cluster (Fig. 1a). The distinctness of the cluster was confirmed with the GRAViTy pipeline (Fig. 1b), which showed that subfamily classifications in the order *Caudovirales* are clustered at the same distance as the new tailed phage family *Ackermannviridae* and as eukaryotic virus families (Aiewsakun et al. 2018; Aiewsakun and Simmonds 2018). A further comparison of all dsDNA viruses using the Phage Proteomic Tree method on the VIPTree server showed that myoviruses, siphoviruses, and podoviruses were interspersed with each other, but SPO1-related phages formed a distinct and coherent clade (Supplementary Fig. S1 available on Dryad). These results clearly indicate that the SPO1-related viruses are distinct and form a cohesive group. Based on this evidence, we propose that this group of viruses represents a new family, and we suggest the name *Herelleviridae,* in honor of the 100th anniversary of the discovery of prokaryotic viruses by Félix d’Hérelle.

### Exploration of the Intrafamilial Relationship

**Figure 2.**
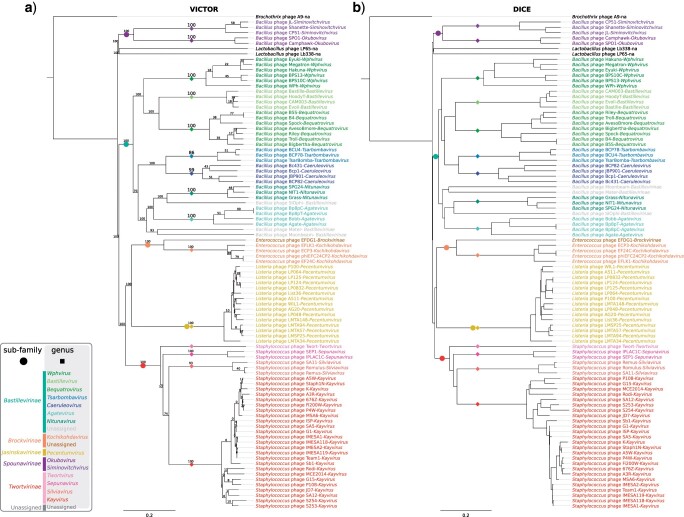
a) VICTOR and b) DICE score trees. The trees were rooted at Brochothrix phage A9. The scale bars represent the calculated distance metric, branch support values at the VICTOR trees were calculated from 100 pseudobootstrap replicates. Genera and subfamilies are delineated with colored squares and colored circles, respectively.

**Figure 3.**
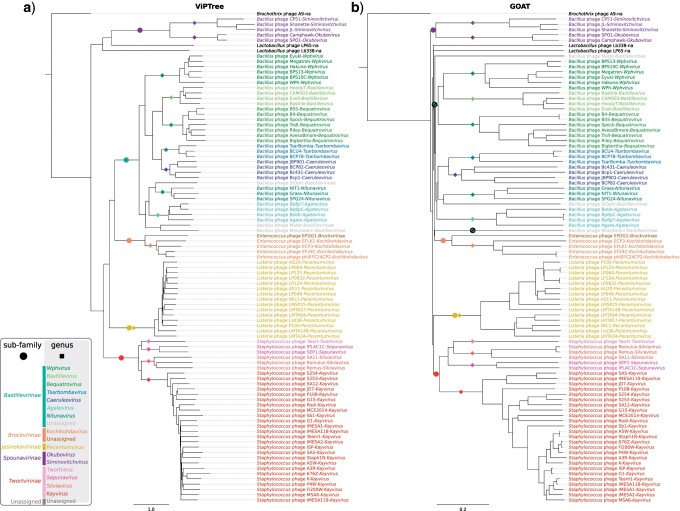
a) Virus Proteomic Tree (VIPTree) and b) GOAT tree. The trees were rooted at Brochothrix phage A9. The scale bar represents the distance metric. Genera and subfamilies are delineated with colored squares and colored circles, respectively.

After delineating the family, we proceeded with the investigation of the relationships between its members. Regardless of the approach applied, we found five clearly-separated clusters interpreted by us as potential subfamilies (Figs. 1b, 2, and 3, Supplementary Figs. S2–S4 available on Dryad, Table 1, Supplementary Table S1 available on Dryad). The first cluster (here suggested to retain the name *Spounavirinae*), groups *Bacillus*-infecting viruses that are similar to Bacillus phage SPO1. The second cluster (*Bastillevirinae*) includes *Bacillus*-infecting viruses that most closely resemble phage Bastille. The third cluster (*Brockvirinae*) comprises viruses of enterococci that are similar to Enterococcus phage $\varphi$EF24C. The fourth cluster (*Twortvirinae*) gathers staphylococci-infected viruses that are similar to Staphylococcus phage Twort. The remaining cluster (*Jasinskavirinae*) consists of viruses infecting *Listeria* that are similar to Listeria phage P100. The classification left three viruses with no genus and subfamily assignment: Lactobacillus phage Lb338, Lactobacillus phage LP65, and Brochothrix phage A9.

Five subfamily-rank clusters can be further subdivided into smaller clades that correspond well with the currently accepted genera (Table 1). The evidence supporting this suggested taxonomic reclassification is presented in the following sections.

### Genome-Based Analyses

The genome-based analyses used to identify close relationships between phage genomes provide powerful information for species and genus demarcation. We performed an all-against-all BLASTn analysis with Gegenees (Ågren et al. 2012), revealing that the genomes of several viruses were similar enough to consider them strains of the same species (they shared $>$95% nucleotide identity, Table 1, Supplementary Table S1, Fig. S2 available on Dryad). We could delineate clear groups with significant nucleotide similarity, proposed as genus-rank taxa, at similarities greater than 50%. Using the BLAST-based phylogenetics framework VICTOR (Meier-Kolthoff and Göker 2017), we were able to confirm that the existing genera form well-supported clades (Fig. 2a).

Similar patterns emerged at the translated nucleotide level when the genomes were analyzed using the tBLASTx-based Dice method (Fig. 2b) (Mizuno et al. 2013). An all-against-all comparison at the translated nucleotide level (tBLASTx) with Gegenees showed an overall low level of similarity (15%) within the newly proposed family and allowed us to start delineating the subfamily level at approximately 25% translated genome similarity (Supplementary Fig. S2 available on Dryad). However, the subfamily boundaries were not always clear using these methods. For example, the members of the *Brockvirinae* subfamily shared 20–25% similarity at the translated nucleotide level with the twortviruses and jasinkaviruses.

### Proteome-Based Analyses

As proteome-based analyses rely on genome annotation, they are sensitive to bias introduced by different annotation methods, and the results of such analyses should, therefore, be interpreted with caution. To mitigate this, we reannotated all genomes with the same automated pipeline as described above (M&M, Supplementary File 1 available on Dryad).

We inferred a Virus (Phage) Proteomic Tree using only the members of the new family to assess its internal structure (Fig. 3a). This showed clearly-defined clusters at the subfamily and genus rank, but revealed longer than expected branch lengths for phages that had very similar genomes, implying that this method should not be used for fine-grained taxonomic classification.

Among 1296 singleton proteins (proteins without recognizable homologues in the analyzed genomes) and 2070 protein clusters defined using the OPC approach, we identified 14 clusters common for all viruses belonging to the new family “*Herelleviridae”* (Table 2, Supplementary Table S2 available on Dryad). Classification of the viral proteins using pVOGs showed that 38 pVOGs were shared between all 93 virus genomes, with 14 pVOGs functionally annotated (Table 2, Supplementary Table S2 available on Dryad). Upon closer inspection of the gene annotations, we found that these analyses might have been confounded by the presence of introns and inteins in many of the core genes. Indeed, many genes of spounaviruses and related viruses are invaded by mobile introns or inteins (Goodrich-Blair et al. 1990; Lavigne and Vandersteegen 2013). These gaps in coding sequences challenge standard gene prediction tools and introduce additional bias in similarity-based cluster algorithms. Because of these insertions as confounding factors, we used a subset of 10 core genes for further phylogenetic analysis.

**Table 2.** Core genes with putative annotated functions identified in all 93 herellevirus genomes

Putative function of the core gene identified$^{a}$	pVOG/OPC ID	Identification method
DnaB-like helicase$^{b}$	VOG0025, OPC6121	OPC, pVOG
Baseplate J-like protein$^{b}$	VOG4691, VOG4644, OPC6132	OPC, pVOG
Tail sheath protein$^{b}$	VOG0067, OPC6142	OPC, pVOG
Terminase large subunit$^{c}$	VOG0051, OPC6160	pVOG
Major capsid protein$^{b}$	VOG0061, OPC6148	OPC, pVOG
Prohead protease	VOG4568, OPC6150	pVOG
Portal protein	VOG4556, OPC6151	OPC, pVOG
DNA primase	VOG4551	pVOG
DNA polymerase I	VOG0668, OPC6097	OPC, pVOG
RNA polymerase	VOG0118	pVOG
Recombination exonuclease	VOG4575	pVOG
Recombination endonuclease	VOG0083	pVOG
Tail tape measure protein	VOG0069	pVOG
Tail tube protein	VOG0068, OPC6141	OPC, pVOG

$^{a}$The full list of protein clusters is available in Supplementary Table S2 available on Dryad (14 core genes identified using OPCs, 38 using pVOGs).

$^{b}$Core genes used in concatenated phylogenetic tree.

$^{c}$Omitted in further phylogenetic analyses due to frequent intron invasion and unclear gene borders.

OPC = orthologous protein clusters; pVOG = prokaryotic virus orthologous group.

### Analysis of Gene Synteny

Viral genomes are thought to be highly modular, with recombination and horizontal gene transfer potentially resulting in “mosaicism” (Juhala et al. 2000; Krupovic et al. 2011). By clustering the herelleviruses based solely on the gene order, we investigated plasticity of their genome structure and potential effects of recombination (Fig. 3b). The clustering results proved comparable with results obtained using sequence-based methods, with almost all viruses clustered according the proposed taxa. The potential exception was Bacillus phage Moonbeam (Cadungog et al. 2015), which showed an inversion of the central part of its genome compared with the other herelleviruses. From this overall picture, we can infer that genomic rearrangements leave a measurable evolutionary signal in all lineages, but do not shuffle genomes of related viruses beyond recognition. Thus, we did not observe the high modularity that might be expected with rampant mosaicism. The lack of considerable mosaicism supports recent findings that, at most, about 10% of reference virus genomes have a high degree of mosaicism (Bolduc et al. 2017a).

### Marker Protein Phylogenies

**Figure 4.**
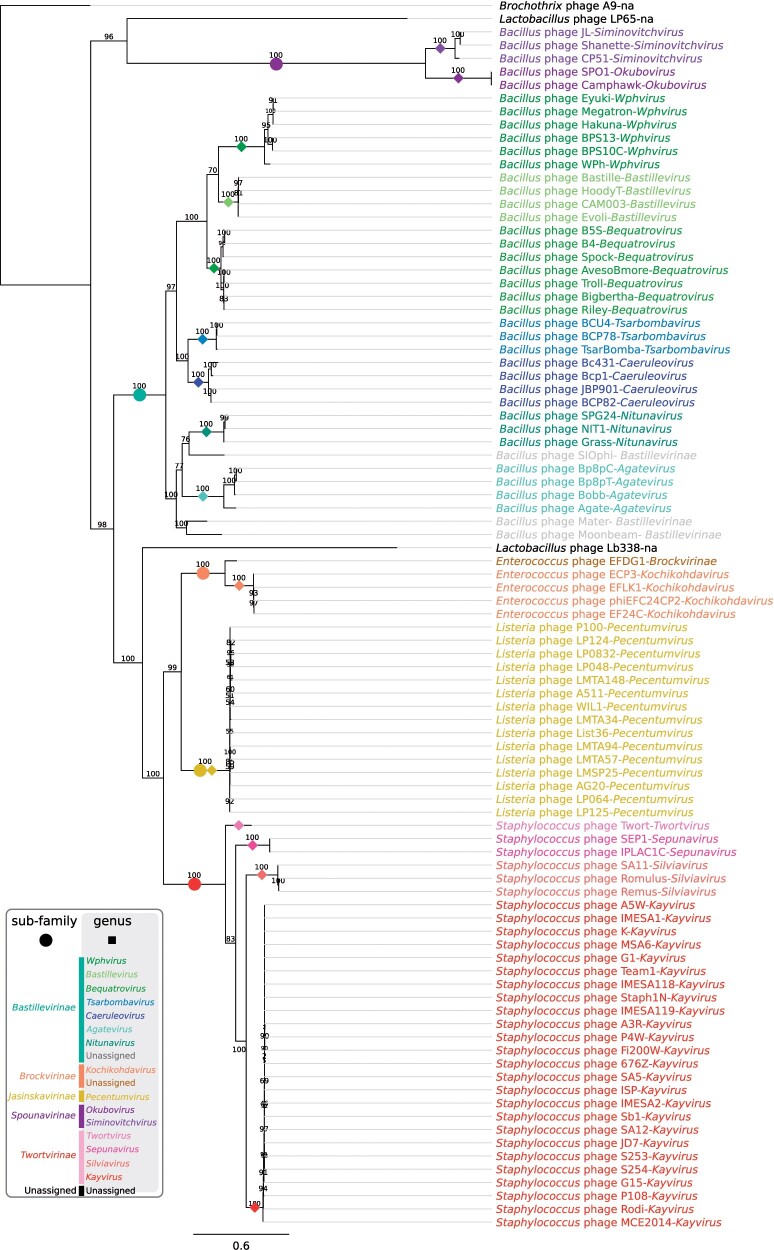
Maximum-likelihood tree based on concatenated alignment of 10 marker proteins generated using IQ-tree. The scale bar represents the number of substitutions per site, branch support values were calculated from 1000 ultrafast bootstrap (UFBOOT) replicates. The trees were rooted at Brochothrix phage A9 to facilitate comparison. Branches corresponding to genera and subfamilies are delineated with colored squares and circles, respectively.

We used the amino acid sequences of concatenated marker proteins identified from the OPC analysis (Table 2) to generate a phylogenetic tree that is able to identify the evolutionary relationships at the genus and subfamily rank within the new family *Herelleviridae* (Fig. 4). This tree supported all proposed new taxa but was unable to differentiate between the different species. Branches representing subfamilies and genera were particularly well-supported (UFBOOT support above 99%). Additionally, nearly all topologies of single marker trees (Supplementary Fig. S3 available on Dryad) fitted well in the suggested taxonomic structure. The only notable deviation from the proposed classification scheme could be found in the Tail tube protein tree (VOG0068–OPC6141, Supplementary Fig. S3 available on Dryad). It shuffled members of the genus *Silviavirus* into the *Kayvirus* clade and also mixed the genera *Nitunavirus* and *Agatevirus* with unclassified phages. This may indicate that the evolutionary signal contained in this marker is insufficient to resolve related genera. Alternatively, the inconsistencies may be explained by the effect of horizontal gene transfer or convergent evolution introducing additional noise in our data. Regardless of the true reason of this inconsistency, it should be stressed that with a small number of available marker loci, additional sources of phylogenetic signal (e.g., whole genome phylogenies) may be necessary to properly interpret any result.

### Comparison of the Results Obtained Using Different Methods

Virus classification methods in general suffer from a low signal-to-noise ratio. This “noise” may be introduced in the data by horizontal gene transfer and differences in mutation rates in different viral lineages. To get a measure of the discrepancies between the methods used above, we calculated the normalized Robinson–Foulds distances (representing the fraction of data partitions that are present only in one of the analyzed trees, Supplementary Table S3 available on Dryad) and created tanglegrams for the visual comparison of topologies (Supplementary Fig. S4 available on Dryad). Trees obtained using different methods differed considerably (normalized Robinson–Foulds metric in range 0.16–0.58) but topological distances between them were comparable to distances between single marker trees (and in most cases smaller, see Supplementary Table S3 available on Dryad). Interestingly, for the herelleviruses, most of the noise becomes averaged at the genus rank, meaning that the grouping at this rank and above remains almost the same regardless of the classification method employed. The only significant discrepancies compared with the proposed taxonomic classification were observed in the GOAT analysis and one single-marker tree (i.e., tail tube protein tree, VOG0068–OPC6141). Both of these deviations concerned a single genus or even unclassified species and they did not follow any commonpattern.

## Discussion

The rapid expansion of phage genomics and metagenomics has left taxonomy behind. There are more than 8000 publicly-available caudovirus genomes, but only 873 have been officially classified by the ICTV (Davison 2017). The remaining genomes are provisionally stashed in the NCBI database within “unclassified” bins attached to the order *Caudovirales* or its associated families (Brister et al. 2015; Adams et al. 2017; Simmonds et al. 2017). One of the main problems is that the level of sequence divergence is so high that it often leaves no detectable sequence similarity between disparate members of the same order. Thus, not a single reliable phage-specific or even *Caudovirales*-specific marker gene could be defined. In addition, a classification system based on a single marker would be highly prone to instances of horizontal gene transfer. Indeed, there is no commonly recognized general phage classification tool and all of the currently used phylogenetic approaches have their critical limitations as described in this study.

For that reason, above the family rank we had to rely on high-throughput network and clustering analyses (vConTACT, GRAViTy, and VipTree) that are capable of discerning the groups of taxa that are comparable, even if phylogenetic signal is sparse. These methods can analyze significant subsets of the viral genomic space in a reasonable time, outcompeting traditional phylogenetic approaches in terms of speed. They are, however, still expensive computationally and need to be recalculated when new data become available (Bolduc et al. 2017b). Moreover, these high-throughput methods do not attempt to model the process that gave rise to the observed data, but rather calculate arbitrary distance matrices from local similarities and use them to define groupings. Thus, the relation between the calculated distance and the divergence time remains unclear and the results of these methods should be taken with a grain of salt, especially in less divergent taxa or at the lower taxonomic ranks.

After defining the new family *Herelleviridae*, we applied a combination of genome and proteome analyses, gene synteny assessments, and multimarker gene phylogenies to establish its internal taxonomic structure. It has to be stressed that the results of most of these methods should be treated as approximations of phylogenic reconstruction. Many of them suffer from similar methodological drawbacks as the abovementioned high-throughput clustering techniques, lacking proper theoretical support of their algorithms. Only the maximum-likelihood analysis of (a) marker sequence(s) allows for rigorous, statistically sound phylogenetic inference under a well-defined model of sequence evolution. Unfortunately, if the number of available marker loci is small, this method becomes vulnerable to the noise introduced by horizontal gene transfer (Davidson et al. 2015). More importantly, this approach is heavily influenced by the gene annotation. This may be a crucial disadvantage as the quality of database records is often debatable and computational reannotation of analyzed genomes does not always yield valid, comparable results.

On the other hand, these drawbacks can be easily circumvented by methods analyzing whole genome sequences (DICE, VICTOR, BLAST). Obviously, they are annotation-independent and mitigate the effects of horizontal gene transfer by averaging the signal across the total genome length. Unfortunately, if the untranslated nucleotide sequence of the virus is used, rapid decay of the similarity should be expected above the genus rank (e.g., Supplementary Fig. S2 available on Dryad). Above that rank, nucleotide sequence similarities were virtually undetectable, but sequence translations (DICE coefficient) or protein sequences (Phage Proteomic Tree) were still considerably similar. Thus, nucleotide sequence-based approaches capture small differences (e.g., silent mutations) between closely related genomes and may be well suited for species and strain demarcation but gradually lose sensitivity with each consecutive taxonomic rank.

To the best of our knowledge, the GOAT algorithm is the only method explicitly aimed at capturing the signal associated with genomic rearrangements in fluid genomes of viruses. Unfortunately, the evolutionary process that is responsible for the observed variations is even less studied than whole genome similarity metrics and we cannot rule out that this algorithm may be disproportionally susceptible to some random rearrangement events. However, it is ideally suited to pinpoint just those kinds of genomic rearrangements and mutations that are missed by other methods. Thus, it can provide unique data on structural dynamics of the studied genomes but in its present form should not be treated as the primary classification tool.

Bearing in mind all the advantages and limitations of the classification tools utilized here, and the convergence of their results for the analyzed taxa, we recommend an “ensemble of methods” approach similar to the one we used as a method of choice for the phage taxonomy. We suggest that future classification efforts should implement at least one well established phylogenetic method (e.g., maximum-likelihood analysis of concatenated marker genes/proteins) and at least one whole genome-based annotation-independent method to account for annotation inconsistencies, rearrangements and mosaicism. Additional approaches may be used, especially if methods of choice produce inconclusive or discordant results but should always be used with regard to their limitations.

All evidence considered, we suggest that the SPO1-related phages should be removed from the family *Myoviridae* and given a family rank. Hence, we proposed establishing a new family *Herelleviridae*, containing five subfamilies: *Spounavirinae* (*sensu stricto*), *Bastillevirinae*, *Twortvirinae*, *Jasinkavirinae*, and *Brockvirinae*, each comprising the genera listed in Table 1. The suggested classification corresponds well with host taxonomy and leaves only 3% of viruses within the new family unassigned. These unassigned viruses may represent clades at the rank of genus or even subfamily that are still undersampled.

Removing spounaviruses from the family *Myoviridae* to form the new *Herelleviridae* family is a major change in phage taxonomy. We envisage this detachment from their original taxon will be followed by abolishment of the* Podoviridae*, *Myoviridae*, and *Siphoviridae* and creation of new “phylogenomic” families, based on current subfamily-rank clades, which will faithfully reflect the genetic relationships between bacterial viruses. In our opinion, these changes are necessary to accommodate the observed diversity of tailed phages. It is worth stressing that this change does not remove the historically established caudovirus morphotypes: myovirids forming virions with contractile tails, siphovirids with long noncontractile tails, and podovirids with short noncontractile ones. Nevertheless, by disconnecting morphotype and taxonomy, related clades can be grouped across different morphotypes. Such an approach would solve the problems of the muviruses that are suggested to be classified in the family “*Saltoviridae*” (Hulo et al. 2015) and potentially the broad set of Escherichia phage lambda-related viruses that are currently distributed among the families *Siphoviridae* and *Podoviridae* (Grose and Casjens 2014). Finally, abolishing the current morphology-based classification of tailed phages will remove the major barrier in classifying phages from metagenomic sequence data.

## Notes

*Dedication: This article is dedicated to Hans-Wolfgang Ackermann, a pioneer of prokaryotic virus electron microscopy and taxonomy, who died on 12 February 2017, at the age of 80. He was involved in the early stages of this study, and his input is dearly missed*.

## Supplementary Material

Data available from the Dryad Digital Repository: http://dx.doi.org/10.5061/dryad.106q6g6.
